# *Apophysomyces variabilis* Infections in Humans

**DOI:** 10.3201/eid1701.101139

**Published:** 2011-01

**Authors:** Josep Guarro, Jagdish Chander, Eduardo Alvarez, Alberto M. Stchigel, Kaushik Robin, Usha Dalal, Hena Rani, Rajpal S. Punia, José F. Cano

**Affiliations:** Author affiliations: Universitat Rovira i Virgili, Virgili, Reus, Spain (J. Guarro, E. Alvarez, A.M. Stchigel, J.F. Cano);; Government Medical College Hospital, Chandigarh, India (J. Chandler, K. Robin, U. Dalal, H. Rani, R.S. Puna)

**Keywords:** Apophysomyces variabilis, mucormycosis, microbiology, cutaneous zygomycosis, Mucorales, fungi, letter

**To the Editor:** The fungus *Apophysomyces elegans* (order Mucorales) is a thermotolerant species that causes severe infections among humans. In contrast to other fungi that cause zygomycosis, which have a worldwide distribution and are rarely found in immunocompetent hosts, *A. elegans* has been reported mainly in areas with warm climates as an emerging pathogen that causes mostly cutaneous infections after injury to the skin (*1*). This fungus was discovered in 1979 (*2*) and until recently was considered the only species in the genus.

A polyphasic study of clinical and environmental strains of *A. elegans,* including analysis of several genes, showed that the genus contained 4 well-characterized species (*3*). Of 16 isolates tested in this study, only 2 from soil in India were *A.* *elegans*. Most of the isolates were *A. variabilis.* The incidence of *A. variabilis* in humans is unknown and difficult to ascertain because most cases had isolates that were not properly preserved. These fungi usually cause necrotizing fasciitis, but rhino-orbito-cerebral or renal infections have also been reported (*1*). Whether these infections are produced by different *Apophysomyces* spp., have different responses to antifungal drugs, or have differences in virulence is unknown.

To assess incidence of *Apophysomyces* spp. in a tertiary hospital (Government Medical College Hospital, Chandigarh, India), which usually receives patients with zygomycosis, a retrospective study was conducted during November 2001–April 2009. Nine patients were identified as having primary cutaneous zygomycosis. For 4 patients, fungal isolates were morphologically identified as *A. elegans.* A description of clinical findings, their management, and outcomes for these 9 patients has been reported (*4*). The 4 isolates were sent to the Universitat Rovira i Virgili (Reus, Spain) for molecular analysis.

The internal transcribed spacer region of these isolates was sequenced and compared with those of type strains of *Apophysomyces* spp. Fungi were identified by morphologic (Figure, panel A) and molecular analysis as *A. variabilis* (99.6%–99.7% sequence identity with sequence of type strain CBS 658.93 [FN556436]). GenBank accession nos. of the 4 isolates are FN813491, FN813490, FN556442, and FN813492.

Another patient was also infected with *A. variabilis* fungi. The patient was a 45-year-old woman with diabetes from Derabassi (Punjab), India, who was hospitalized because of swelling in her right breast and blackening of overlying skin. A diagnosis of right breast gangrene was made. Therefore, local debridement of the swelling was conducted, and tissue samples were tested by microbiologic culture and histopathologic analysis.

A KOH wet mount showed broad aseptate hyphae with right-angled branching. The fungal isolate was tentatively identified as *A. elegans*. Histopathologic analysis confirmed a diagnosis of zygomycosis. The patient was treated under local anesthesia by debridement of infected tissue and some of the healthy surrounding tissue (Figure, panel B). However, an antifungal regimen could not be given because she had disturbed renal function. Her condition deteriorated, septicemia was observed, and she died from sudden cardiac arrest on the sixth day after admission. The fungal isolate was also identified as *A. variabilis* (98.9% identity, GenBank accession no. FN556443).

Although most cases of infection with *A. variabilis* fungi have been reported in India (*5*), infections with this fungus may have a wider distribution. A recent study demonstrated that this species represented 0.5% of fungi of the order Mucorales isolated from clinical samples in the United States (*6*). Furthermore, a high mortality rate and the fact that most of these infections involve otherwise healthy patients make this a serious infection.

The number of infections with *Apophysomyces* spp. is underestimated because these fungi do not usually sporulate on standard fungal culture media used in clinical laboratories. These fungi require special nutrient-deficient growth medium (Czapek agar), a high temperature in comparison to other human pathogens (37°C–42°C), and prolonged incubation (7–10 days) (*7*).

A difference in mortality rate was observed when we compared patients in our study (80%) with those reported by Chakrabarti et al. (*5*) (28.5%) in India, even though treatment was generally similar, i.e., local débridement and amphotericin B. Other differences in our study were that the infection in 4 patients was preceded by intramuscular injection, and these 2 patients had diabetes mellitus.

In conclusion, *A. variabilis* is an emerging pathogenic fungus that can cause rapid and fatal infections in humans. As more isolates of *Apophysomyces* fungi become available, molecular typing studies must be conducted to better understand the epidemiology and distribution of different *Apophysomyces* spp.

**Figure:**
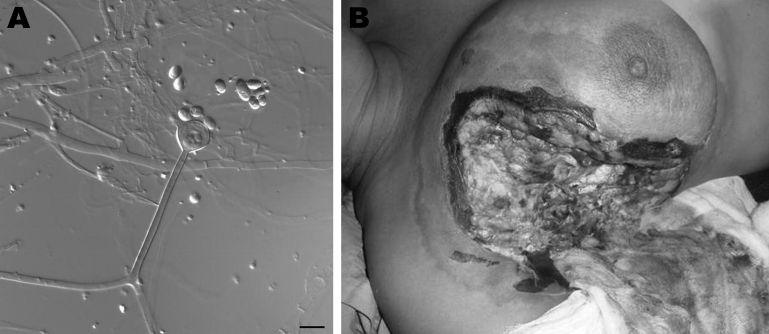
A) Sporangiophore (center) and sporangiospores of *Apophysomyces variabilis* fungi. Scale bar = 10 μm. B) Clinical manifestations in a woman infected with *A. variabilis* fungi in the upper part of the chest and the breast. A color version of this figure is available online (www.cdc.gov/EID/content/17/1/134-F.htm).
